# The Effects of Integrated IT Support on the Prehospital Stroke Process: Results from a Realistic Experiment

**DOI:** 10.1007/s41666-019-00053-4

**Published:** 2019-05-23

**Authors:** Magnus Andersson Hagiwara, Lars Lundberg, Bengt Arne Sjöqvist, Hanna Maurin Söderholm

**Affiliations:** 1 PreHospen - Centre for Prehospital Research, Faculty of Caring Science, Work Life and Social Welfare, University of Borås, SE-501 90 Borås, Sweden; 2 Department of Electrical Engineering, Biomedical Signals and Systems, Chalmers University of Technology, SE-412 96 Gothenburg, Sweden; 3 PreHospen-Centre for Prehospital Research, Faculty of Librarianship, Information, Education and IT, University of Borås, SE-501 90 Borås, Sweden

**Keywords:** Stroke, Decision support, EMS team, Prehospital process

## Abstract

Stroke is a serious condition and the stroke chain of care is a complex. The present study aims to explore the impact of a computerised decision support system (CDSS) for the prehospital stroke process, with focus on work processes and performance. The study used an exploratory approach with a randomised controlled crossover design in a realistic contextualised simulation experiment. The study compared clinical performance among 11 emergency medical services (EMS) teams of 22 EMS clinicians using (1) a computerised decision support system (CDSS) and (2) their usual paper-based process support. Data collection consisted of video recordings, postquestionnaires and post-interviews, and data were analysed using a combination of qualitative and quantitative approaches. In this experiment, using a CDSS improved patient assessment, decision making and compliance to process recommendations. Minimal impact of the CDSS was found on EMS clinicians’ self-efficacy, suggesting that even though the system was found to be cumbersome to use it did not have any negative effects on self-efficacy. Negative effects of the CDSS include increased on-scene time and a cognitive burden of using the system, affecting patient interaction and collaboration with team members. The CDSS’s overall process advantage to the prehospital stroke process is assumed to lead to a prehospital care that is both safer and of higher quality. The key to user acceptance of a system such as this CDSS is the relative advantages of improved documentation process and the resulting patient journal. This could improve the overall prehospital stroke process.

## Background

Prehospital emergency care refers to the work provided by care personnel to patients with acute health problems outside of the hospital, most often in response to emergency calls and dispatch to patients’ homes, accident scenes or other locations. Emergency medical services (EMS) clinicians are key actors in this work. They diagnose and provide care on scene and, if needed, transport patients to a medical facility [1]. Quality of care, patient outcomes and thus patient safety is heavily depending on EMS clinicians’ ability to make fast and accurate decisions based on structured assessments. This can be challenging in the prehospital domain due to uncertain or unexpected circumstances, safety issues and access constraints, in combination with different levels in training, practices and skills among prehospital care personnel. Furthermore, some care processes are highly time critical. One example of a time-critical process is when being dispatched to a patient with stroke symptoms.

Stroke is a common condition which affects 15 million people worldwide each year. 4.5 million of these will die from the stroke and among survivors, 50% will have disabilities after 6 months [2]. The EMS is an important link in the stroke process: care provided by EMS clinicians is the first point of access to care for the majority (> 70%) of patients with stroke symptoms [3]. The prehospital stroke process is complex, involving several time-critical steps and actions. Examples include the following: establishing a field diagnose; determine a time for symptom onset; using protocols for treatment decisions; information exchange and communication with emergency room (ER) and/or neurologists; and producing accurate and reliable documentation.

The full stroke process (the time from symptom onset to definitive care) is determined by and dependent on several factors. One of the most crucial factors is delay time, which can be divided into *patient delay* and *system delay*. Patient delay refers to the time from a patient’s first symptoms to when they make the first contact with the health care system (e.g. when calling an ambulance). System delay is the time from this first contact until the final diagnosis is established and critical treatment initiated [4]. There are indications that a comprehensive prehospital assessment and early identification of patients with time-critical conditions can decrease the total system delay time even though this includes increased prehospital time [5]. Studies have revealed that patients with stroke benefit from early contact with the health care system, e.g. using EMS rather than seeking care from primary care or using their own means for transportation to an ER. Early recognition of stroke symptoms has a positive effect on minimising system delay time, e.g. recognised already at the dispatch centre [6, 7], by EMS clinicians [8] and through early notification to the receiving hospital [9]. There are however challenges related to assessments and decision-making in field settings, such as accurately diagnosing a stroke. Two previous studies have shown that about 35% of stroke diagnoses (later determined at the hospital) are in fact missed by EMS clinicians [10, 11].

Today, the support provided to EMS clinicians to manage patients usually consists of a combination of cognitive tools or expert systems such as paper-based protocols or guidelines, and phone consultations with attending ER physicians or (for stroke symptoms) neurologists. As discussed by Andersson Hagiwara et al. [12] and Söderholm [13], there are however problems with these tools and practices, in the sense that they are not always suitable for the task or compatible with the EMS work process. As an example, EMS clinicians have to use several different paper-based protocols, guidelines, triage systems and medical record documentation sheets or systems, when managing a patient. In complex time-critical prehospital processes such as management of patients with stroke symptoms, it is neither effective nor practical to assemble, find and use all these different sources of critical information, potentially in combination with performing continuous documentation, and using communication gadgets and other tools. Hence, the process could benefit from an integrated computerised decision support system (CDSS) enabling access to medical history, computerised process support (e.g. assessments rules and process guidelines), opportunities to send and receive information to/from medical support and the possibility to document directly in a patient’s digital prehospital medical record.

Much of the previous work on CDSS has been done within clinical contexts such as hospitals and primary care, and is reported in a number of systematic reviews [14–17]. These reviews indicate that a CDSS improves patient assessment, increases compliance to guidelines, and as a result has a positive effect on quality of care and patient safety. The largest effect on quality of care is when the CDSS can be used in the direct patient contact [18], e.g. by using handheld devices which is reported to have better effect on care processes than stationary technology [19]. Furthermore, the use of a CDSS can standardise clinical decision-making and as a consequence give patients more equal assessment and treatment [18]. One challenge is however that clinical/hospital healthcare settings are very different from prehospital settings in terms of what type of work that is done, the environments, safety factors and people working there. Hence, different healthcare environments require different types of CDSS.

The few studies of CDSS in the prehospital care context indicate that using a CDSS can improve the patient assessment process [20] and increase compliance to guidelines [21], but that the systems need to be carefully tailored to the specific user group and their needs—otherwise technology support could have harmful effects [22]. If the CDSS, e.g. is too difficult to use or cumbersome to set up, it might add unnecessary time to treatment, delay care or not be used in a correct or efficient way. As discussed in a systematic review by Bano et al. [23], there is a strong correlation between user involvement and system success. User participation has been identified as one of the most important factors for system success [24], while failing to understand the intended users has repeatedly been reported as a reason to failed system development projects [23, 25–27]. Hence, when designing these types of systems, it is crucial to work in multidisciplinary teams (e.g. including both designers, developers and healthcare professionals), and involve users early in the design process using realistic and contextualised approaches to evaluate the system use and its impact on work processes as well as outcomes.

In this study, we present the results of an evaluation of a CDSS prototype for prehospital stroke managements. The prototype is called PrehospIT and was manufactured by the company Ortivus. It enables interoperability between different systems so that prehospital personnel can access as well as enter information to get continuous decision and documentation support throughout the whole prehospital care process via one single system/interface. We have strived to create a realistic, simulated evaluation environment where real users (EMS clinicians) perform their work in very similar ways to what they usually do, using the CDSS prototype throughout an entire ambulance mission, and then share their perspectives on the advantages and challenges of this specific technology.

### Study Aims

The aim of this study is to explore the effects and user perceptions of the PrehospIT CDSS on the prehospital process of managing patients with stroke symptoms. The aim pertains to (1) the impact on work process and performance, and 2) user perceptions of benefits and problems when using the system. The aims have been further operationalised as follows:

1. The PrehospIT CDSS’s effect on work process and performance, with respect to:

- EMS teams’ clinical performance
- EMS teams’ compliance to process recommendations
- Prehospital system delay time
- Work distribution within the team

2. Perceptions of using the PrehospIT CDSS with respect to:

- EMS clinicians’ self-efficacy
- Advantages and challenges regarding compatibility and design of the PrehospIT CDSS

The PrehospIT CDSS is intended to be used throughout an ambulance mission, from dispatch to finalising the patient record. This use situation spans several physical locations, and includes different activities such as assessments, decisions and communication, and both using/accessing/gathering and sharing/recording/sending information from and to other stakeholders, locations and actors within the overall healthcare system (see Table 1 for an overview of phases and activities). From an evaluative point of view, this introduces several challenges, which makes real-life evaluation impractical and risky. Therefore, we base our evaluation on a realistic, contextualised simulation approach [28].

**Table 1:** Prehospital phases, activities and PrehospIT CDSS functionality

Phase	Activities and locations	Functionality and support provided by the PrehospIT CDSS
Receiving the call	• *Finding the address*; the first priority is to find the address and best route to the patient • *Assess possible risks*; information from the dispatch centre is used to assess safety. Is the address known? Is there information of drug abuse or violence or hazardous environment? • *Gathering supplementary information*; is there more information from dispatch centre? Calling the patient or relatives • *Make plans*; what is the possible scenario? Who in the team is responsible for what? Which equipment should be brought to the patient? Is there a need for more resources? What do guidelines say?	• Possibility to access the patient’s chart and medical history • Access treatment guidelines
Arriving at the scene	• *Assess possible risks* by gathering visual information • *Scene assessment*; gathering and assessment of information such as weather conditions, light conditions and information of social condition • *First impressions*; assessment of information such as body position, skin colour, body constitution, breathing and body movement	• Possibility to make notes about first impressions
On scene assessment and treatment	• *First survey*; assessment and treatment of Airway, Breathing Circulation, Disability and Exposure (ABCDE) • *Second survey*; focused patient history, focused assessment, vital parameters and triage • *Clinical decision-making*; level of urgency, general treatment decisions, level of care. • *Interventions*; on scene treatment	• Guidance to perform surveys and assessments in a structured manner • Functionality to support continuous documentation and recording of assessment results • Decision support based on findings • Treatment and process protocols, e.g. mNIHSS for stroke
Transport decision and departure	• *Transport decisions*; decisions including leaving the patient at home with self-care advice, transport to primary care, transport to nearest hospital or transport to specialist hospital • Re-evaluation; ABCDE • *Decisions on transport techniques*; best techniques for transport to the ambulance?	• Re-evaluation and/or re-assessment based on same support as above • Notification to hospital and/or neurologist with option to send field record/assessment and mNIHSS-score
En route assessment and treatment	• Re-evaluation; ABCDE • *En route assessment*; continued focused patient history and assessment • *En route treatment*; continued treatment • *En route communication*; communication with receiving hospital by phone or text message	• En route assessment and treatment based on same support as above based on same support as above • Notification to hospital and/or neurologist with option to send field record/assessment and mNIHSS-score
Handover	*• Arrival*; preparing the patient for hospital handover • *Handover report*; structured handover report • *Documentation*; documentation according to local routines and systems (paper based/digital) • *Preparation for new mission*; cleaning or replacement of used equipment • *Reflection*; critically analysing clinical events and outcomes.	• The use of the system forms the base for a digital prehospital record. The record can be use as member notes in the verbal handover report • After the handover the EMS team can finish the digital prehospital record and send it to the hospital patient record database.

## Methods

### Study Context

A typical ambulance mission can be divided into a number of defined phases [12, 29, 30]. Earlier simulation studies of ICT technology for prehospital care [20, 22, 31] have only focused on one specific phase of the prehospital process, such as on-scene assessment and treatment. It is reasonable to assume that every type of new tool or healthcare technology affects different processes in the overall care chain in different and diverse ways [32]. Therefore, it is crucial to investigate not only specific tasks or outcomes in isolation but also consider the system’s impact on the overall work process [33]. To be able to study the use and the effects of a CDSS in relation to the whole prehospital care process, it is important that every phase is included in an evaluation. The evaluation in the present study covers all these phases. The phases and how the PrehospIT CDSS supports the activities in each of these are outlined in Table 1.

#### The PrehospIT CDSS System

**Fig. 1:**
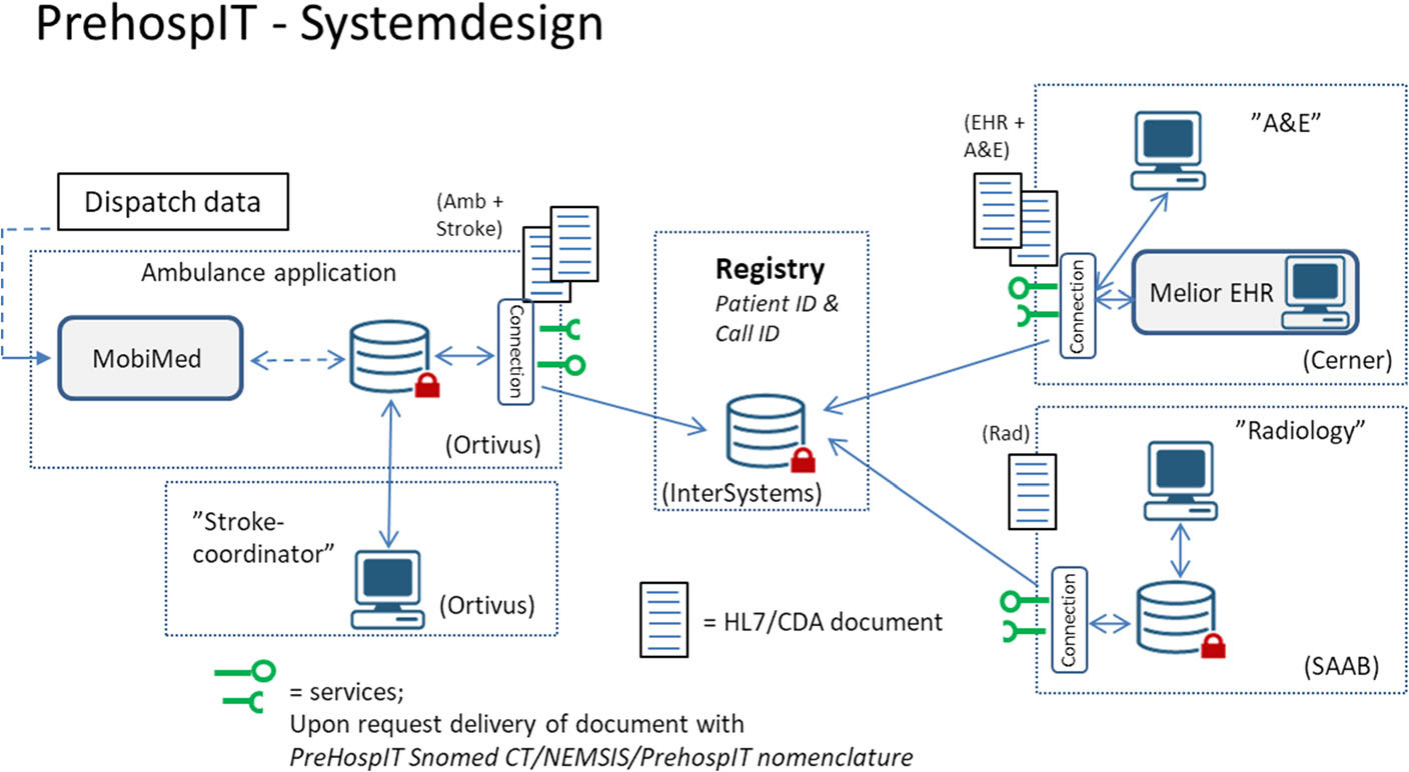
PrehospIT system design

The technical set-up of the PrehospIT system consisted of products and components from four different providers (Ortivus, Cerner, SAAB and InterSystems). Together, they represented the following: one ambulance IT-solution (MobiMed, by Ortivus); one electronic health record system (Melior, by Cerner); a PrehospIT specific radiology department client (by SAAB); a PrehospIT specific stroke coordinator client (by Ortivus); a PrehospIT specific alarm and event (A&E) client (by Cerner); and one registry (HealthShare, by InterSystems). As part of the PrehospIT project, all parties had agreed upon standards, protocols and a system set-up to enable flexible interchange of data between their respective standard products and/or PrehospIT specific components. A central component in the overall system solution was the Registry, which kept track of where information regarding a specific patient (ID) was added and/or stored. Requests to the Registry made from any component in the system set-up, allowed direct retrieval of information related to the specified patient ID from any other source in the system. This information was formatted as a HL7 CDA document,^1^ with content coded in accordance with a mutual PrehospIT standard utilising NEMSIS,^2^ Snomed CT^3^ and a proprietary protocol to handle what was not covered by the existing standards. This system set-up made it possible to support the entire acute stroke chain with real-time input from existing electronic patient records (EPRs) or other data sources available, including real-time field input via the ambulance application evaluated in the present study. It also enabled the various clients (A&E, Radiology and Stroke coordinator) provided by various companies to retrieve information in real time from any source including the ambulance. Prior to the simulations, the set-up was technically tested and verified in a connectathon workshop (Fig. 1).

### Study Design

**Fig. 2:**
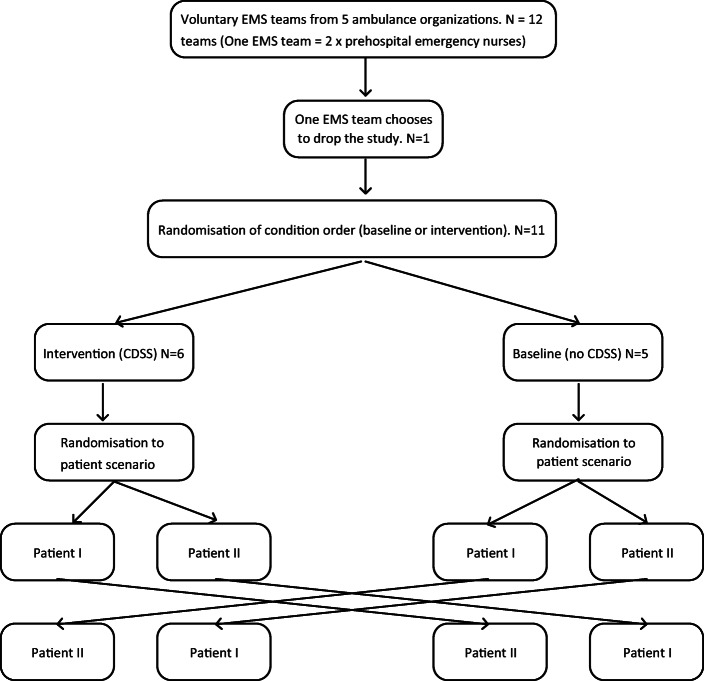
Experimental design flow-chart

The study follows the realistic evaluation paradigm [34]. This means that equal attention is paid to the context (the prehospital work process), and to the mechanisms (activities, interactions and tools) within the context. According to Pawson and Tilley [34], mechanisms are always embedded in particular contexts and it is hence necessary to understand effects together with the context, on evaluation outcomes. The study compares the clinical performance between EMS teams using a CDSS and EMS teams using usual paper-based process support. The participating EMS teams were randomised to four different conditions: to start with using CDSS and patient scenario one, start with CDSS and patient scenario two, start without CDSS and patient scenario one, and start without CDSS and patient scenario two. The study design was inspired by the framework of Kannampallil et al. [33] for evaluating clinical cognitive activities in complex real-world environments. See Fig. 2 for study design flowchart.

The teams worked through two simulated patient care scenarios based on patients exhibiting stroke symptoms. Patient 1 was an elderly man with confusion and difficulties to stand up (stroke mimic). The team was dispatched to the person’s home where he lived with his partner. For patient 2, the team was dispatched to a 40-year-old man found on the floor at his workplace (a real estate agent’s office) by a co-worker. The patient exhibited difficulties to speak and asymmetrical neurological findings. Participating teams were working through the whole ambulance mission outlined in Table 1, including dispatch, driving, arrival, on-scene care, patient interaction, interaction with relatives and bystanders, transport, handover at hospital and final patient chart documentation.

### Participants and Setting

The participants were all active EMS clinicians from four different ambulance organisations in Sweden. EMS clinicians in Sweden include (1) ambulance nurses with the main medical responsibility in the EMS team, and (2) emergency medical technicians (EMTs) with a shorter education. This formation somewhat resembles the Anglo-American system, with paramedics and EMTs. Since 2005, all ambulances in Sweden must be staffed by at least one registered nurse [35] and an EMS team can consist of either two ambulance nurses or one ambulance nurse and one EMT. The teams in our study were recruited through enquiries distributed to managers of EMS organisations asking if any clinicians in their organisation were interested to participate in the study. Twelve teams consisting of 24 EMS clinicians announced an interest to participate. Three weeks before the experiment, they received information via email about the study, the simulation and the CDSS. Eleven teams showed up to participate in the experiment. On arrival at the facility, participants received additional information regarding the study and their participation, and signed an informed consent form. The team was then introduced to the CDSS and was able to interact with and explore the system for approximately 30 min. They also received a 20-min introduction to the simulation, the patient simulator and the EMS bag and its equipment. The participating clinicians (15 = men, 5 = women, 2 = n/a) had a mean age of 41 years (SD = 8.2), averaging 11 years of EMS work experience (SD = 7.9) (Table 2). Based on our experience from the field, this reasonably mirrors the current spread in gender and experience level (no national statistics available).

**Table 2:** Participant characteristics

Team/participant	Sex		Year of birth	Years in EMS
Team 1	P1	♂	1979	6
	P2	♀	1978	13
Team 2	P3	n/a	n/a	n/a
	P4	♂	1972	8
Team 3	P5	♂	1958	27
	P6	n/a	1971	15
Team 4	P7	♀	1992	4 months
	P8	♂	1979	7
Team 5	P9	♂	1989	2
	P10	♂	1969	14
Team 6	P11	♀	1980	3.5
	P12	♂	1968	25
Team 7	P13	♂	1974	17
	P14	♂	1967	19
Team 8	P15	♂	1970	15
	P16	♂	1965	24
Team 9	P17	♂	1983	8.5
	P18	♀	1977	13
Team 10	P19	♂	1987	8 months
	P20	♀	1972	1
Team 11	P21	♂	1974	8
	P22	♂	1975	12

The experiment was conducted in a simulation facility at an ambulance organisation in south west Sweden. The simulation platform is a part of a research project (SAREK) [36] with the aim to contextualise the prehospital work process using a serious game-inspired approach to accomplish better simulation [37]. The platform has been tested for immersion [38] and has been found superior to traditional simulation where some phases in an ambulance mission are excluded and with lower equipment and environmental fidelity. Using a contextualised simulation, platform was found to affect immersion positively and thus contributes to an improved simulation experience [28].

### Data Collection and Analysis

#### Performance

All scenarios were video recorded in their entirety, from dispatch to hand-over. These recordings were used as a basis for measuring teams’ performance during scenarios.

The performance measure was based on two parts: *time* and *clinical performance*. Time included timestamps of the video recordings that was used to calculate, e.g. on scene time, transport decision and hand-over. Clinical performance was analysed from the video recordings by a panel of six experienced ambulance nurses. To do this a validated instrument, global rating scale for the assessment of paramedic clinical competence (GRS) [39] was used together with a protocol covering the regional prehospital guidelines for stroke assessment and treatment procedures. The GRS instrument was used to evaluate seven competence items plus overall clinical performance (see Table 3). The performance for each item was graded on a 7-point Likert scale ranging from unsafe (1) to exceptional performance (7). In addition to this, the raters used the regional guideline to count performed interventions (performed or not), record whom in the team performing the interventions (1st or 2nd EMS clinician) and to record times for different interventions and phases. Examples of recommended interventions according to the regional prehospital guidelines are first survey and second survey based on the assessment process described in the advanced medical life support (AMLS) course [40] including assessment and treatment of vital functions (airway, breathing, circulation, disability, exposure); structured patient history^4^; and stroke-specific focused assessment. The most important step in the prehospital stroke assessment is the graded neurological examination with the instrument modified National Institutes of Health Stroke Scale (mNIHSS) [41]. It is based on the hospital instrument NIHSS [42] where the neurologist has to decide if the patient can be transported to the nearest hospital with access to computer tomography (CT), bypassing the ER, and treatment with thrombolysis or direct transport to specialist centre with thrombectomy resources. The scale includes 11 items representing different assessments, scored from 0 to 2, 0 to 3 or 0 to 4. The mNIHSS instrument starts with assessment of level of consciousness, followed by assessment of gaze, visual fields, motor functions of left and right arm, motor functions of left and right leg, and assessment of sensory, language and neglect. The mNIHSS instruments used in the participating ambulance organisations are to some extent different from the original instrument (neglect is excluded and thus has a different max score). Maximum score in the original instrument is 31, with scores between 2 and 5 indicating minor stroke and scores > 6 indicating a major stroke involving major brain vessels. In addition to the mNIHSS instrument, the focused assessment described in the regional guidelines includes electrocardiography (ECG) transmissions to hospital and measurement of serum glucose.

**Table 3:** Theoretical constructs used in data analysis (based on technology acceptance and innovation adoption)

Construct	Definition
Relative advantage	Benefits for stakeholders such as patients, physicians or EMS clinicians. Potential impact on different parts of the care process. Usefulness of the PrehospIT CDSS.
Compatibility	How well the PrehospIT CDSS works in relation to current work practices. Disadvantages for stakeholders/study participant. Situations or contexts where the PrehospIT CDSS would not work or be useful.
Complexity	If the PrehospIT CDSS is perceived as cumbersome or complicated to use or to learn how to use. Dimensions related to interacting with the system and its interface and physical design.

The six raters (2 = female, 4 = male) were all experienced and active ambulance nurses, with a mean experience of 10.2 years (SD = 4.2) and a mean age of 40.0 years (SD = 7.6). Two of the raters had previous experience of using the GRS instrument for rating clinical performance. The analysis session started with instrument training, where the raters together watched one experiment scenario and discussed the rating using the GRS instrument and the additional guideline protocol. After the training session, the raters were independently rating one common scenario. From these results, an inter-rater reliability (IRR) analysis was performed. The IRR was assessed using a two-way mixed, consistency, average-measures intra-class correlation (ICC) [43]. An ICC value over 0.60 is considered a good IRR. The resulting ICC among the six raters was in the excellent range, ICC = 0.83, indicating a high degree of agreement. After the IRR analysis, the video recordings were randomised between the six raters so each rater was rating 3 to 4 videos independently (22 in total). The raters had not been involved in the design of this particular study, and also rated a number of other scenarios from another experiment involving simulations and devices (including IT). Thus, they were not aware of the differences in any of the experimental conditions they rated, and it was not possible for them to determine if a team was using the CDSS or not. The CDSS was built into the ordinary monitoring equipment used in all of the simulated scenarios and the raters were not able to see the specific actions the EMS teams did on the computer screen.

For sample size calculation, the research group used data from a recent simulation experiment [28] and scored the video recordings from the experiment with the GRS instrument. The sample size calculation was based on results from overall clinical performance. The mean difference between the two conditions in that experiment was 0.83 and the standard deviation 0.83. With an α-level of 0.05 and power of 80%, the calculation gave a minimum of 10 subjects to be able to reject the H_0._

To compare differences in time and clinical performance between groups, paired *T* test was used for data on interval level and for nominal data we used the McNemar test. A *p* value of < 0.05 was considered significant in all statistical tests. For all other data, descriptive statistics were used. All statistical analyses were performed using the statistical software program SPSS 21.0 (SPSS Inc., Chicago, IL).

#### Self-Efficacy

The social cognitive theory of self-efficacy refers to a person’s judgement of their capability to perform a certain task [44]. Self-efficacy contributes to future task performance in the sense of skill development and skill retention as it stimulates certain behaviours. High levels of self-efficacy lead to more efforts to solve a challenging problem, and thus additional skills, while low levels of self-efficacy reduce and/or slow down skill development. If the tools used to solve a problem (e.g. technology) are badly designed, disruptive, or lack work-fit, this might harm users’ self-efficacy and thus their future performance [45]. For this study, we used a self-efficacy instrument (a post-questionnaire) designed to examine different types of skills (e.g. decision-making, assessment, awareness) in a prehospital mission. EMS clinicians indicated how confident they were that they could perform each task on a 5-point Likert scale (1 = strongly disagree; 5 = strongly agree). Since no self-efficacy questionnaire for the prehospital care work process was available, we developed questions based on Bandura’s [46] recommendations and in consultation with prehospital care researchers, instructors and practitioners. A Wilcoxon signed-rank test was used to determine differences between participants working with and without CDSS.

#### User Perceptions of the PrehospIT CDSS

Post-interviews were conducted after the team had worked through the two scenarios. A semi-structured interview guide was used, based on concepts pertaining to technology acceptance [47] and innovation adoption [48]. Questions included teams’ perceptions of whether the CDSS was easy to use, easy to learn, how compatible it was with their current way of working, what advantages using the system provided compared with their current practice, and what problems and challenges they experienced when using the system.

Post-interviews were analysed in two steps, first bottom-up to identify initial broad themes (e.g. “positive impact”, “negative use experience”, “documentation”, “current practice”). These were then analysed top-down according to the theoretical constructs (Table 3) and where applicable also organised in an affinity diagram. The affinity diagram is a tool used in, e.g. human-computer interaction and contextual design [49] to visualise themes and their relationships in relation to the work and tasks that a system or design intends to support.

## Results

### The CDSS Effect on EMS Teams Clinical Performance

#### GRS Scoring of Overall Clinical Performance

The GRS scoring among the six raters showed statistically significant differences for two items. There was improvement in score of the item “patient assessment” when the team was using the CDSS (*t* = − 2951, *p* = 0,014) mean score increasing from 4.09 ± 0.94 to 5.27 ± 0.79. There was also a significant difference in the item “decision-making” (*t* = − 3131, *p* = 0,011) with increase in GRS score from 4.73 ± 0.65 to 5.36 ± 0.50 when using the CDSS. There were no significant differences in the other GRS items between when teams were using CDSS or not (Table 4), all of them however exhibiting positive change tendencies except for “resource utilisation” and “procedural skills”.

**Table 4:** Results from GRS rating of teams working as usual compared with working with the PrehospIT CDSS

Variable	Mean difference	SD	*t*	95% confidence interval	*p*	Effect size (Cohen’s *d*)
Situation awareness Teams’ ability to consider and integrate environmental, scene, resources and patient condition into the overall management plan.	+ 0.18	1.08	0.56	− 0.91–0.54	0.59	0.28
History gathering Teams’ ability to effectively gather a patient history according to the clinical situation and level of urgency.	+ 0.25	1.06	0.82	− 0.92–0.42	0.43	0.40
Patient assessment Teams’ ability to select and perform a physical exam and investigation of signs and symptoms.	+ 1.18	1.33	2.95	− 2.07 to − 0.29	0.01*	1.37
Decision making Teams’ ability to select an appropriate and safe management plan.	+ 0.63	0.67	3.13	− 1.09 to − 0.18	0.01*	0.97
Resource utilisation Teams’ ability to identify and use resources effectively to accomplish goals and maximise care.	− 0.18	1.33	0.45	− 0.71–1.07	0.66	0.18
Communication Teams’ ability to clearly exchange information within the team, patient and bystanders for optimal patient care and team effectiveness.	+ 0.54	0.93	1.96	−1.17–0.08	0.08	0.90
Procedural skill Teams’ ability to complete procedural skills effectively and in accordance with standards.	0.00	1.67	0.00	−1.12–1.12	1.00	0.0
Overall clinical performance Overall judgement based on the seven items.	+ 0.55	1.04	1.8	−1.2–0.15	0.11	0.67

*Significant difference; *+*, higher score with use of CDSS, *SD*, standard deviation

#### The CDSS Effect on EMS Teams’ Compliance to Process Recommendations

The scoring of the number of accomplished assessments and treatments interventions as described in the prehospital guidelines showed that the CDSS had a positive impact. The teams accomplished significantly more interventions in the first survey (mean differences + 1.82, *t* = 4.8, *p* = 0.01) including assessment and treatments of airway, breathing, circulation, disability and head-to-toe assessment when using the CDSS. There was also a significant difference in stroke-specific assessment as described in the prehospital guidelines where teams accomplished more assessments (mean difference + 2.27, *t* = 3.6, *p* = <0.001) when using the CDSS. For the total number of assessment and treatment interventions, using the CDSS led to significantly more interventions being performed compared with working as usual without the support of a CDSS (mean difference + 6.0, *t* = 5.10, *p* = <0.001) (Table 5).

**Table 5:** Results of performed enquiries and interventions comparing EMS teams working as usual with when working with CDSS

Variable	Mean difference	SD	*t*	95% confidence interval	*p*	Effect size (Cohen’s *d*)
First survey (rec interventions *n* = 5)	+ 1.82	1.25	4.8	− 2.66 to − 0.98	0.01*	1.71
History gathering (rec questions *n* = 14)	+ 1.45	2.91	1.66	− 3.41–0.50	0.13	0.72
Focused assessment (rec enquiries *n* = 12)	+ 2.27	1.95	3.6	− 3.59 to − 0.96	< 0.01*	1.60
Vital parameters (rec parameters *n* = 6)	+ 0.45	0.82	1.84	− 1.01–0.10	0.10	0.72
Interventions (rec interventions *n* = 5)	+ 0.45	1.43	1.05	− 1.42–0.51	0.32	0.46
Total interventions (rec interventions *n* = 42)	+ 6.00	3.90	5.10	− 8.62 to − 3.38	< 0.01*	1.74

*Significant difference; *+*,higher score when using the CDSS; *SD*, standard deviation

#### The CDSS Effect on Prehospital System Delay Time

The team using the CDSS was spending approximately 4 min longer time compared with the team working as usual without a CDSS. The use of the CDSS also increased the time of notification to neurologist (Table 6). The higher number of performed interventions in the CDSS group can partly explain the reason for this system delay. Number of interventions/min was 0.83 in the CDSS group compared with 0.74/min in the control group (mean difference = + 0.09/min, *t* = 2.52 *p* = 0.03).

**Table 6:** Critical times for teams using CDSS in compare with teams without

Variable	Mean difference (mm:ss)	SD	*t*	95% confidence interval	*P*	Effect size (Cohen’s *d*)
Total scenario time	+ 04:32	0:20	2.38	− 08:48–− 00:17	0.04*	0.78
Total time with patient (on scene and in ambulance)	+ 03:49	06:29	1.95	− 08:11–00:32	0.08	0.68
On scene time	+ 02:51	05:07	1.85	− 06:16–06.44	0.09	0.64
Time with patient in ambulance before departure	+ 01:04	02:18	1.46	− 02:43–00:34	0.18	0.52
Time to notification to neurologist	+ 04:16	05:18	2.55	− 08:03–− 00:28	0.03*	0.75

*Significant difference; *+*, increased time with use of CDSS, *SD*, standard deviation

#### The CDSS Effect on Work Distribution

Using the CDSS had a tendency to change the EMS teams’ work distribution. When teams were using the CDSS, the 2nd EMS clinician performed more assessments and treatment interventions compared with when they worked without the CDSS. The results were not statistically significant but showed a consistent tendency towards that using a CDSS might change how work is distributed within a team (Table 7).

**Table 7:** Number of interventions performed by 1st clinician and 2nd clinician with and without CDSS

Variable		Performed by 1st clinician (valid %)	Performed by 2nd clinician (valid %)	*p* value
1st Survey				
	Baseline	25 (80.6)	6 (10.9)	0.75
	CDSS	32 (74.4)	11 (25.6)	
Anamnesis				
	Baseline	32 (68.1)	15 (31.9)	0.80
	CDSS	29 (65.9)	15 (34.1)	
Vital parameters				
	Baseline	28 (62.2)	17 (37.8)	0.08
	CDSS	21 (42.9)	28 (57.1)	
Stroke specific assessment				
	Baseline	43 (76.8)	13 (23.2)	1.0
	CDSS	46 (75.4)	15 (24.6)	
Total				
	Baseline	128 (71.5)	51 (28.5)	0.07
	CDSS	126 (64.6)	69 (35.4)	

Frequencies and *p* value produced with McNemar’s test

### Self-Efficacy

When rating their capability to perform different tasks in a prehospital mission on a 5-point Likert scale, EMS clinicians rated their ability high. Overall mean including all 30 self-efficacy items was 4.21 in the CDSS condition and 4.24 in the condition when clinicians worked without a CDSS. Only small differences were present between the two conditions. A Wilcoxon signed-ranks test detected mixed statistical significant results (at *p* < .005). Table 8 is showing an excerpt of the 7 of the 30 items in the full self-efficacy questionnaire exhibiting the largest positive and negative differences (range + 0.20 to − 0.32) between the CDSS condition and the condition with no CDSS.

**Table 8:** Excerpt of results from self-efficacy survey

*I am confident that I can…*	Without CDSS	With CDSS	Mean diff	*p*
Perform focused surveys?	4.00	4.20	+ 0.20	.157
Determine a preliminary diagnosis?	4.00	4.20	+ 0.20	.102
Continuously monitor and provide treatment and care to the patient during transport?	4.32	4.19	− 0.13	.046
Inform and communicate with the patient?	4.20	4.00	− 0.20	.206
Give notice to receiving hospital in a concise and structured manner?	4.27	4.05	− 0.23	.096
Communicate and collaborate with the others in the ambulance team?	4.64	4.36	− 0.27	.058
Distribute roles?	4.36	4.05	− 0.32	.052
Overall self-efficacy (based on all 30 items)	4.24	4.21	− 0.03	

### User Perceptions of Using the CDSS During an Ambulance Mission

#### Relative Advantage

The main advantages of using the CDSS in relation to current practices were related to the following: access to information, process support and documentation and information sharing.

**Fig. 3:**
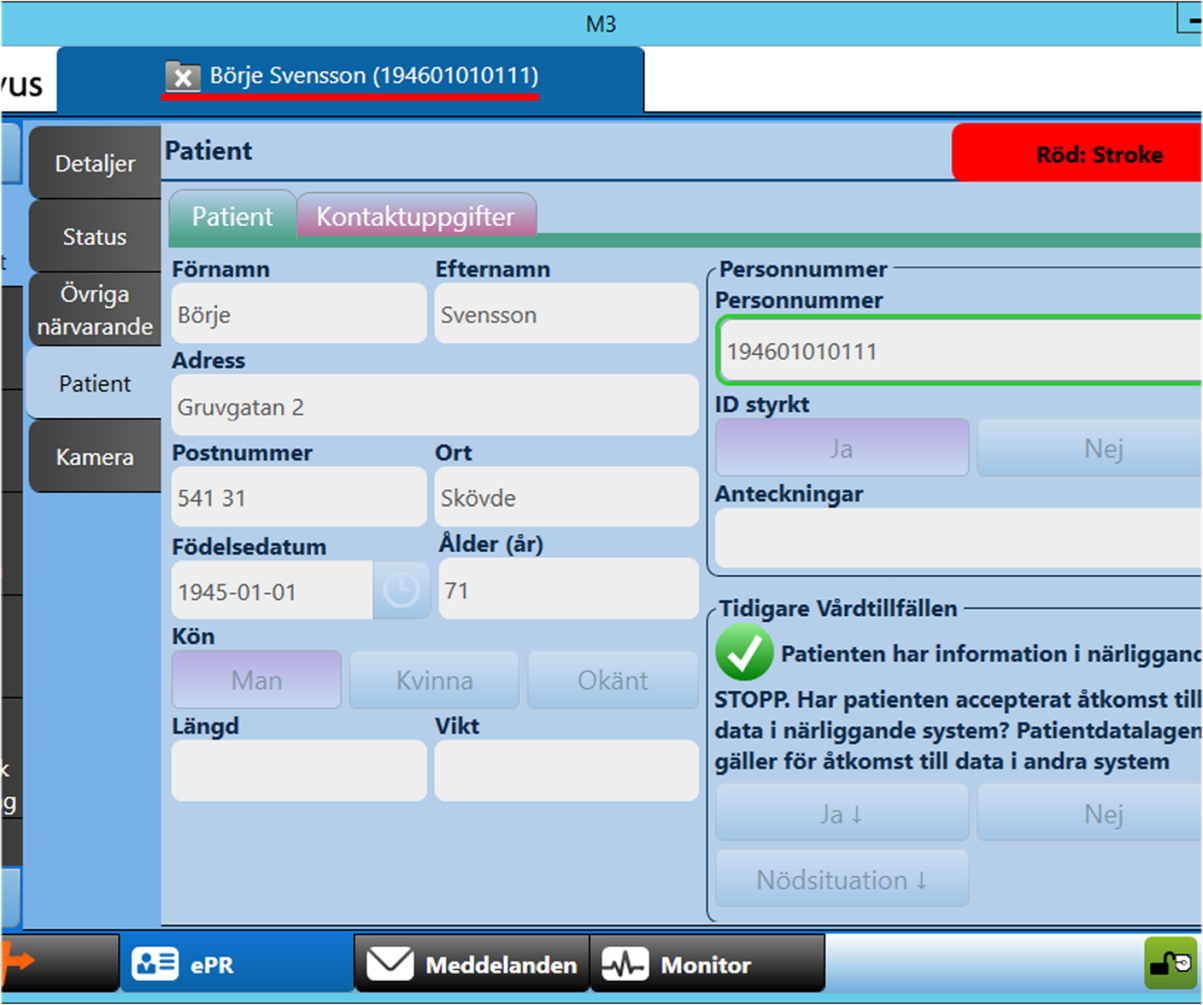
Screenshot of CDSS providing information during dispatch

Getting access to information about the patient already during dispatch (Fig. 3) was found beneficial in terms of being able to prepare and plan and to get more background information.

*“Already during dispatch I checked the records of past illnesses, because we had the personal information and everything. So already at that stage you get a good insight of what the patient may have suffered or what is important to know, if there are any previous diseases or not.”* (Team 9)

Some participants however worried that this would narrow their focus and impede them from seeing all possible options: *“... the danger in that is so that you don’t arrive with a clear preconception about the patient.”* (Team 3).

The process support functionality was appreciated by participants. It helped them to remember to perform all assessments according to their guidelines, and that the results of these assessments were recorded simultaneously via the system.

*“…if you get a little insecure, ‘have I forgotten something?’ or gets stressed, then I can see if I've checked it and can I go back and do it so that I include all [items], in order to make a good assessment.”* (Team 8)

*“…you do not miss anything, but you'll follow the manual in a better way. I think it can be a great support.”* (Team 7)

*“The [paper] checklist we have in the cars, that you have to flip through to find, [that flipping] disappears since this is digital, I think that was smooth.”* (Team 11)

Some participants found the process support to be more valuable for cases more in the grey zone than more extreme cases, such as the simulated stroke scenarios:

*“... it’s far from all [patient cases] that are so clear. (…) here [in this scenario] there were sky high points and one hour from onset... often, I think it’s a little more grey zone. Here you might do very well without any support at all – you have to go towards a stroke.”* (Team 11)

*“Abdominal pain, the simple ones where you're not really sure. Not these extreme things. If you have a patient, say priority two [triage], that isn’t super urgent in any way, then you have the time to use the system the way I think it's supposed to be [used].”* (Team 7)

One participant explains that some of the process support e.g. for differential diagnosis is more helpful than support to work through basic initial assessment such as ABCD:

*“We are thinking ABCD in all situations (...) that is in our role, as obvious as it is when you turn the steering wheel while driving a car. On the other hand, I think that the differential [diagnosis] part is quite interesting (...) it was quite cool anyway, that the more [boxes] you checked the more it [the computer] tried to figure out what this could be about, as a reminder to us that this might perhaps not be a stroke, it may be "this". I think that is good, because if you have not had that thought yourself, the computer will help you to think that thought.”* (Team 11)

There was however participants who were hesitant, or thought their colleagues would be hesitant, to some aspects of the process support:

*“…that a computer would tell us what kind of patient we have, I think some may be sceptical to that. We do better than a computer, I think. Because there is always a scepticism about everything that is new. But to get a patient’s journal that writes itself, that would have been rejoiced.”* (Team 11)

Hence, the strongest advantage using the PrehospIT system was related to documentation, in particular in relation to the patient record.

*“It feels like this is really the future. Especially if it [the CDSS] is made so that what is written becomes the patient’s journal, so that we don’t have to, like we do now, write one [ambulance journal] that is to be transferred and then you have to re-do one [write a new one at the hospital] (---) Then you can actually spend more time [on one journal], now a lot is missed when you have to write two, that is our big problem.”* (Team 8)

*“[Using the CDSS] is not double documentation in the same way as when you're sitting with a paper first... it’s getting more in real time as you document.”* (Team 7)

**Fig. 4:**
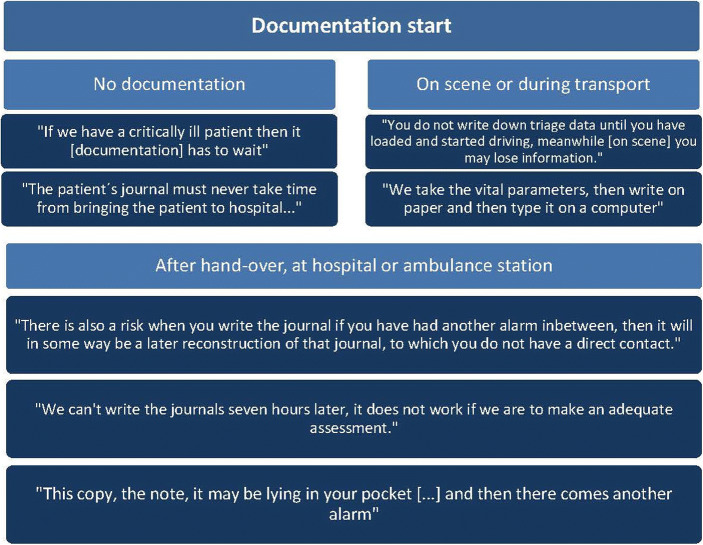
Illustration of participants’ accounts of the current documentation process

These statements are not surprising: current documentation practices suffer from a number of problems. The affinity diagram (Fig. 4) illustrates different variations and challenges in the current documentation process that emerged in our data.

As Fig. 3 shows, the main part of the final documentation for the patient chart is often, after being started on scene or during transport, done after handover. Depending on type of EMS system, the final ambulance chart is entered into a digital system either at the hospital or the ambulance station based on unstructured notes or paper journal charts. The ongoing documentation provided by the PrehospIT CDSS would introduce immense benefits with respect to accuracy and patient safety but also lessen work-related stress. Participants report that the PrehospIT CDSS would provide a number of documentation and information sharing benefits:

*“... [benefits for] patient safety and work environment, because it is a stress to go around with those records in your pocket. It feels really bad; you feel that you have not done your job”* (Team 2)

*“... you can send it [the chart] right away, then [the doctor] can read 20 minutes before we get in [...] and they will receive continuous updates”* (Team 4)

*“The most valuable is that the times are recorded... exactly everything. Because today, our times do not make sense when you come in with a stroke patient. ‘Well how long ago was that?’ You do not even know what time you arrive at the patient, so you look at the dispatch time, and then you try to plot a little bit, ‘that might have taken a quarter.”* (Team 1)

In order to (1) get this type of accurate and reliable documentation output and (2) to get the process support such as differential diagnoses suggestions or stroke-specific guidelines/support, continuous input of information into the CDSS while assessing and treating the patient is required. Doing so, raised issues related to the CDSS’ compatibility with current ways of working.

#### Compatibility

Although the CDSS was found to be compatible content-wise with the prehospital process—in terms of containing relevant and useful information and providing relevant types of support, it was not fully compatible with the work process, especially when it came to using it to input information during patient care:

*“…but you also have to do that while you see the patient. (---) When you start that care chain, get the score, send the alarm. If you compare that with our paper, there are five points and what we do on these trigger the alarm. Here these points were to be filled in, I thought that was very difficult, especially with the computer.”*

All participants raised concerns about difficulties to focus on the patient while using the CDSS. This was their most prominent concern with the CDSS.

*“I felt that now I have to set my brain to computer mode, then I have to switch back to patient mode again”* (Team 5)

Furthermore, using the system was not compatible with the current roles and distribution of work within the team. Many teams expressed confusion about their roles and work division when using the CDSS even though some tried to prepare for this during dispatch:

*“It was a little trouble in how to use the computer support. We had some discussion on the way out: [if] one person talks to the patient and the other will check and supplement with something, how to make that work as smoothly as possible. Or would it be the one who does it [talks to the patient] also should work the computer? That felt a little bit, we felt we do not really know how to do it. (---) Though it's something you have to try out.”* (Team 8)

*“We had decided that you would mark [things done on the computer] but once we got to the patient so… when I looked at you... ‘but this will not work, maybe I’ll take the computer instead.”* (Team 3)

This also included difficulties to coordinate their work, sometimes due to lack of awareness where in a process or protocol the other person “were”:

*“When standing next to him, and he uses the computer, the cooperation gets harder as well. Otherwise, we are with the patient and talking. Now he has to sit and concentrate there [at the computer] and I’ll have to wait a bit here and ask "will we do that now?” Then he is elsewhere in his mind according to the computer, where I am not. Collaboration is complicated when [doing this]. So I felt that it took longer because of it, because the cooperation did not really flow as quickly.”* (Team 5)

As several teams suggest, there is a need to rethink how work is divided so that responsibilities for documentation and focusing on the patient is in balance within the team:

*“I felt that I was busy with it because I did not get it completely. Otherwise when you work, one cares for the patient, and the other notes the parameters. Now I would sit with it [the computer], I did not get all the information from the patient. So, really, it's the other caregiver who's should sit and type the data? (---) We talked a bit about it earlier, that now maybe [2nd EMS] gets a bigger role to take control since I’m going to check [the boxes in the computer]. Actually, one should have done the opposite, that [2nd EMS] would have recorded what I reported to that person, if I was to be 1*^*st*^*EMS anyway. If I am responsible for the care, then I want to have that contact with the patient and then someone else will have to fill in those parameters [in the computer].”* (Team 6)

If done correctly, using the CDSS could improve the coordination and mutual awareness within the team:

*“But I think the flow will be better if the 1*^*st*^*EMS takes the vital parameters, reports, speaks aloud, then you get a communication where you [2nd EMS] gets a picture [of what's happening]”* (Team 3)

Overall, the means to input information using the CDSS does not add any advantages compared with current paper-based practice; the output is however seen as superior to what is produced today in terms of accuracy, information sharing and patient safety.

#### Complexity

All teams thought the PrehospIT CDSS provided relevant support and correct and relevant content. They found it easy to learn and that the introduction they received before the simulation was sufficient. Several commented however, that they needed to use it more in order to feel comfortable with using it, e.g. through simulations.

*“... even though you were allowed look at it before [the scenario], the questions did not come up until you really sat down [facing] all the buttons”* (Team 9)

*“I do not think that even if we had added 40 minutes to the introduction, I do not think it would have been better... you have to use it for real a little bit.”* (Team 11)

Hence, the issues identified above with respect to difficulties to focus on the patient while using the CDSS, and thus the need to re-coordinate the work within the team, are connected to the complexity of the interface design: *“The system needs to be easier to use in order to not take away the focus from the patient”* (Team 1).

The interface of the Prehospit CDSS was perceived as messy and difficult to navigate in. Negative aspects expressed by several teams included *“too much scrolling”, “difficult to get an overview”, “difficult to navigate and find where content is located”, “too many menues and sub menues”.*

Participants mentioned several ways in which the interface design could be improved:

*“An alternative to browsing, for example, if you want to use the checklist would be that as soon as I enter a response to a question item then the next one is automatically showed. Then you keep an eye on the screen and ask [the patient] that question”* (Team 1)

**Fig. 5:**
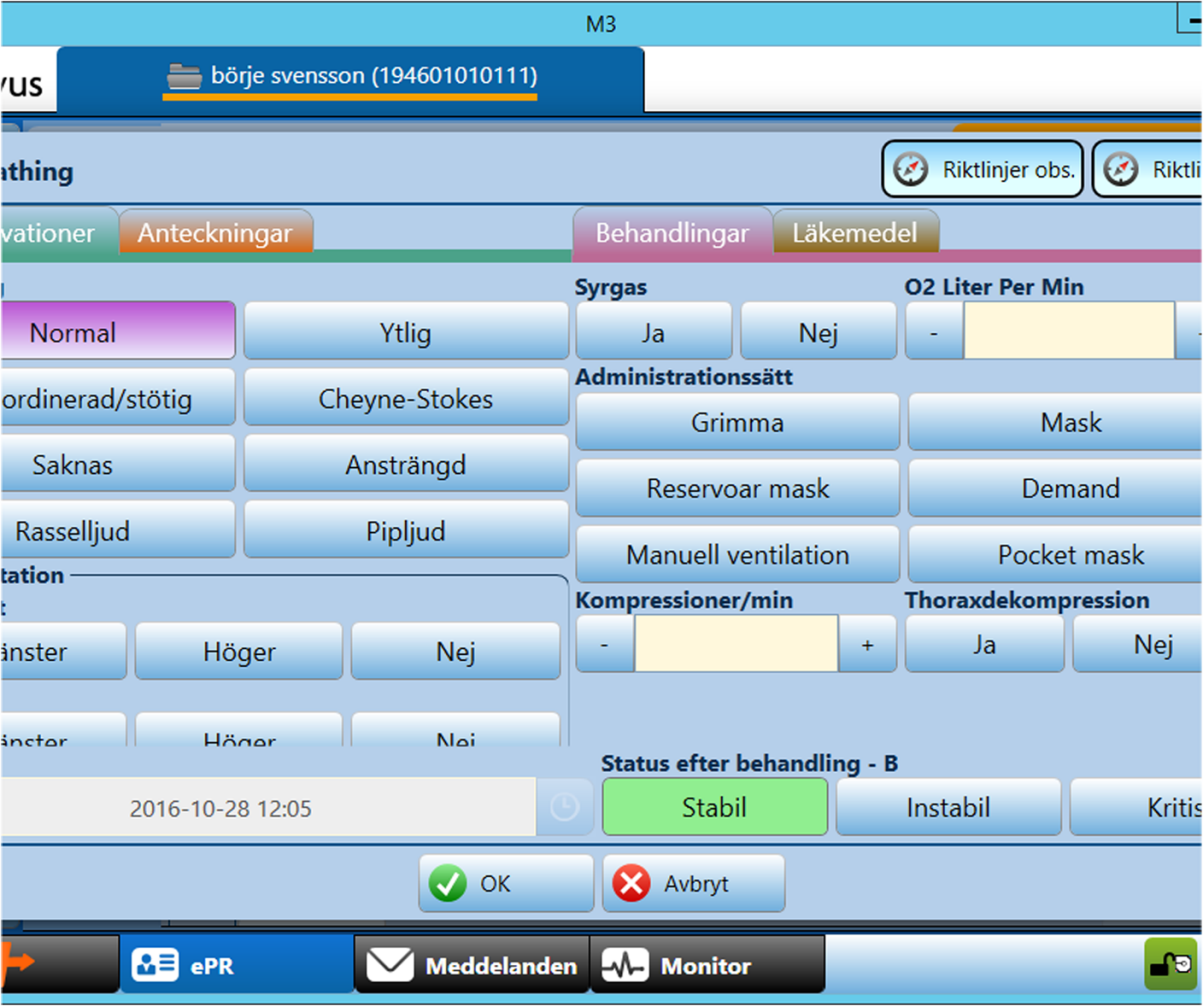
Screenshot of one of the interfaces for recording information in the CDSS

Furthermore, several participants mentioned features they missed, although these actually were available in the system. Hence, the interface design lacks visibility, in terms that it sometimes wasn’t obvious what was possible to do. As Figs. 3 and 5 show, there are boxes for writing comments in free text but participants still asked for the option to do so, not only use boxes and set alternatives.

When considering the reported difficulties to use the system, it is however important to keep in mind that this was the first time participants used it, and that it will take some more time to learn. As participants reflect:

*“It’s the first [time] and then of course, it feels a little bit messy. Should I do the same thing tomorrow, I might have learned the ropes a bit more.”* (Team 6)

When it comes to hardware-related aspects such as interacting with the PrehospIT CDSS, this is done through entering text via a keyboard, and through using a pen/pointer to scroll, push buttons and cross checkboxes etc. Participants were hesitant to this and found it difficult and potentially even more difficult if being under stress or while driving:

*“And even worse if you're in the car while driving, then you jump around [when trying to scroll].”* (Team 8)

Some suggested that the physical design was too cumbersome and felt outdated and that more current technology should be used, e.g. that support touch-based interaction, similar to an iPad or smartphone.

*“…a menue like a cell phone’s [menue] that you can enlarge instead of having a touchpad pointer (---) people are used to cell phones. (---) A smaller tablet but still the same functions (---) Yes, like an iPad, not this large thing.”* (Team 3)

There were also issues raised regarding hygiene:

*“Like this screen with these keys, it cannot be kept clean. You should have something that's pretty flat if it's supposed to work.”* (Team 6)

Overall, the system was perceived as easy to learn and use. Complexity issues include interface design and physical design that currently should be better adapted to the prehospital care context.

## Discussion

When teams were using the CDSS, significant improvements were found in the two GRS items referring to patient assessment and decision-making. These findings are not surprising. It is during the on-scene assessment and treatment an ambulance team makes most decisions and it is also in this phase of a mission that they have the least support [29]. One earlier study [12] revealed that the physical format of current paper-based guidelines and protocols makes explicit use difficult in the critical on-scene phase. To be able to effectively use guidelines and protocols during direct patient contact is considered as an important feature for safe healthcare [18].

The increased compliance to process recommendations found in the present study is in line with previous research. In a previous simulation study [20] on-scene assessment and treatment of two simulated patient cases, a CDSS did significantly increased EMS teams’ compliance to assessment recommendation and anamnesis. That CDSS was partly based on the same platform as the PrehospIT system and had a similar interface. One reason for the increased compliance compared with paper-based guidelines is probably the explicit, step-wise use of guidelines prompted by the CDSS. When using the CDSS, the EMS clinicians take part of the process recommendation in the same process and system interface as working through their patient assessment. The teams using the CDSS spent a longer time on-scene. Previous work [20] reports similar results when a CDSS was used in simulations, although when later evaluated in a clinical study, [21] the effect of increased on-scene time however disappeared. This result suggests that the increased on-scene time induced by the PrehospIT CDSS could be partly explained by the short introduction of the system in connection to the experiment. However, although a longer prehospital time may be negative for patients with stroke, it is important to emphasise that if the extended time is caused by a more accurate assessment, the total time to final care may be shortened.

As previous work [15] discuss, new technology such as a CDSS requires some type of incitement or relative advantage [48] compared with current ways of doing things in order to be accepted and effectively used. The key to acceptance for the PrehospIT CDSS is the continuous real-time documentation of assessments and treatment results generating a complete patient journal accessible for the ER. This has several advantages: (1) Information is recorded in real time, close to the first patient contact rather than hours or even days afterwards. It is reasonable to assume that this would lead to more reliable patient data. (2) It addresses the issue discussed by Söderholm et al. [50] that when physicians at the ER or later in the healthcare chain make crucial medical decisions, they seldom have access to the prehospital patient journal or information from scene such as mechanism of injuries, physical findings or any prehospital treatments [51]. The prehospital information is crucial for preparation and for determining further course of treatment; however, a large amount of this is lost during hand over [52] and thus not accessible for the physician [53]. Being able to send a journal draft during transport gives the ER team more information early and thus time to prepare, and makes the process less dependent on a verbal handover report. This is especially important in time-critical processes such as stroke.

Overall, our results suggest a small impact of this CDSS on participants’ self-efficacy. This is important because badly designed systems might harm self-efficacy and thus future performance [45]. The largest differences in self-efficacy between conditions are found in items regarding assessment and establishing a preliminary diagnosis (participants’ self-efficacy was higher for these items in the CDSS condition) and in items related to patient interaction, team coordination and communication, where participants’ self-efficacy was lower in the CDSS condition. These results mirror the GRS rating as well as post-interview results, in terms of that the CDSS provided support in assessment and diagnosis, but interfered with the patient interaction and teams’ work distribution process.

While the positive impact and relative advantages of the PrehospIT CDSS are several, the interface design was perceived as cumbersome and quite difficult to use during an ongoing care process. In particular, participants found it difficult to handle the constant move between focusing on the CDSS and the patient. The two modes can be attributed to the reasoning styles/systems described in dual process theory [54]. According to this theory, people use two systems of reasoning: system 1 which is fast, unconscious and automatic, and system 2 which is slow and analytical. The two systems are both in use during clinical reasoning decision-making (CDRM), but in emergency medicine, system 1 is dominating the process [55]. This system is more prone to decision bias, e.g. clinicians making premature decisions and not finishing assessment processes [56]. It is more common in urgent situations where patients show symptoms of failure in vital functions than in more stable situations when there is time for more analytical reasoning (system 2) [57, 58]. In prehospital care, system 2 reasoning is supported by cognitive tools, e.g. guidelines, protocols and algorithms [29]. This has been referred to as system 2-by proxy, and includes elements of algorithmic reasoning and ruling out the worst case scenario [59]. As suggested by our results, the easy, integrated access to guidelines and process support provided by the PrehospIT CDSS prompts the clinician to use a more analytical style of reasoning (system 2). This leads to a more complete and structured assessment of the patient. But, as the participants in the present study express, the constant switching between the patient (e.g. quickly deteriorating patient prompting a fast and more automatic system 1 response) and system/computer mode was a cognitive challenge impacting the process and the team dynamics. This emphasises the importance of a physical and intuitive interface that is truly designed for the intended users and their specific context in order to minimise cognitive load when using the system [60]. Content wise, the interface should provide a clear overview of the work process while not being too crowded; and also support easy interaction, e.g. touch-based interaction or voice interaction similar to the interaction technologies used in smartphones and tablets [60].

As several of our results suggest, both the team work process and patient interaction was affected by the CDSS. Changes in team work process was reflected in the negative GRS rating score for the item resource utilisation (Table 4); changes in how work was distributed within the team (Table 7); a negative mean difference in self-efficacy items related to team and patient interaction (Table 8); and participants’ accounts of difficulties to focus on the patient and to coordinate the work within the team while simultaneously using the CDSS. It is reasonable to assume that the issues related to cognitive challenges, teamwork and patient interaction to some extent (in addition to the design issues previously mentioned) also was due to that this was the first time participants used the CDSS. Even though everyone thought preparation time was sufficient, in order to be fully comfortable with the system, they need to work with it and apply it continuously to real situations. These results also highlight the importance of agreeing on how to coordinate a team work process and roles, e.g. who should perform exams, interact with the patient and use and operate the CDSS system. The use of contextualised simulation to explore the use of the CDSS was highly appreciated by all teams. Hence, we strongly recommend that when a new tool such as a CDSS is developed and later implemented, training in context, e.g. contextualised immersive simulation proposed by Engström et al. [28] is required. This approach also helped us to explore use and impact of the system for different phases of the process, e.g. early documentation as a mechanism for improved coordination between field and ER and identify areas for improvement regarding the system design.

## Limitations

In this evaluation, we included the entirety of the prehospital work process in the simulated scenarios. One limitation of our approach is, however, that we do not include any inquiries/data from physicians with respect to perceptions of information sent from the scene or ambulance via the CDSS, such as preliminary patient chart and mNIHSS assessment done on scene. To be able to include that in the study, a simulated hospital set-up would have been required, in addition to coupling every team with a corresponding number of consulting neurologists and/or ER physicians. This was not feasible for us to do in terms of time frames, resources and availability of physicians. It is however crucial in future studies to also include the hospital side as well as perspectives from physicians, that is—what do the people at the hospital do with the information sent from the scene?

Another limitation is that the analysis is done on team level only. Both our quantitative and qualitative results point towards changes in work distribution and potentially work roles when using the PrehospIT CDSS. This will be further analysed and reported in a subsequent publication.

In this study, we used paper-based processes as baseline since this currently is the most common practice in Swedish prehospital care. This is a potential limitation since other geographical regions might have other practices and/or use other types of CDSS.

## Conclusions

Using CDSS might improve patient assessment, decision making and compliance to process recommendations. The key to user acceptance is the advantages of improved documentation and the resulting patient journal. This could improve the overall prehospital stroke process and bridge the information gap between ambulance and hospital. Negative effects of the CDSS include increased on-scene time and a cognitive burden of using the system, affecting patient interaction and collaboration with team members.

## Data Availability

The main data set consist of video recordings of EMS teams in action. Parts of the data set can be provided on request. Please contact first author.

## Abbreviations

CDSS: Computerised decision support system service
ER: Emergency room
ECG: Electrocardiography
EMS: Emergency medical service
GRS: Global rating scale
CRDM: Clinical reasoning and decision making

## Footnotes

1 The HL7 version 3 Clinical Document Architecture (CDA®), http://www.hl7.org/implement/standards/product_brief.cfm?product_id=7

2 The National Emergency Medical Services Information System, https://nemsis.org/

3 SNOMED CT The Global language of healthcare, https://www.snomed.org/snomed-ct

4 Structured patient history: signs and symptoms, onset, provocation or palliation, quality of the pain, region and radiation, severity, time, allergies, medications, past illnesses, last oral intake, events leading up to present illness
